# An RNA Interference Tool to Silence Genes in *Sarcoptes scabiei* Eggs

**DOI:** 10.3390/ijms23020873

**Published:** 2022-01-14

**Authors:** Deepani D. Fernando, Pasi K. Korhonen, Robin B. Gasser, Katja Fischer

**Affiliations:** 1 Infectious Diseases Program, Cell and Molecular Biology Department, QIMR Berghofer Medical Research Institute, Brisbane, QLD 4006, Australia; Deepani.Fernando@qimrberghofer.edu.au; 2 Department of Veterinary Biosciences, Melbourne Veterinary School, The University of Melbourne, Parkville, VIC 3052, Australia; pasi.korhonen@unimelb.edu.au (P.K.K.); robinbg@unimelb.edu.au (R.B.G.)

**Keywords:** RNA interference (RNAi), gene silencing, *Sarcoptes scabiei*, parasitic mite, egg stage

## Abstract

In a quest for new interventions against scabies—a highly significant skin disease of mammals, caused by a parasitic mite *Sarcoptes scabiei*—we are focusing on finding new intervention targets. RNA interference (RNAi) could be an efficient functional genomics approach to identify such targets. The RNAi pathway is present in *S. scabiei* and operational in the female adult mite, but other developmental stages have not been assessed. Identifying potential intervention targets in the egg stage is particularly important because current treatments do not kill this latter stage. Here, we established an RNAi tool to silence single-copy genes in *S. scabiei* eggs. Using sodium hypochlorite pre-treatment, we succeeded in rendering the eggshell permeable to dsRNA without affecting larval hatching. We optimised the treatment of eggs with gene-specific dsRNAs to three single-copy target genes (designated *Ss-Cof*, *Ss-Ddp*, and *Ss-Nan*) which significantly and repeatedly suppressed transcription by ~66.6%, 74.3%, and 84.1%, respectively. Although no phenotypic alterations were detected in dsRNA-treated eggs for *Ss-Cof* and *Ss-Nan*, the silencing of *Ss-Ddp* resulted in a 38% reduction of larval hatching. This RNAi method is expected to provide a useful tool for larger-scale functional genomic investigations for the identification of essential genes as potential drug targets.

## 1. Introduction

Scabies is a highly significant skin disease of mammals caused by the burrowing, microscopic parasitic mite *Sarcoptes scabiei*. In a direct life cycle, female mites invade the skin and lay many eggs. From these eggs hatch larvae, which develop to nymphs and differentiate into male or female adult mites within ~2 weeks [1]. Throughout the mite’s lifecycle, the eggs are the only ‘amplification’ stage.

Scabies is one of the commonest dermatological conditions of humans, with >150 million cases globally, representing a burden of 1.5 million years lived with disability (YLDS), predominantly in resource-poor, disadvantaged communities. Estimated prevalence of human scabies vary from 0.2% to 71.4% in different areas of the world [1,2,3]. Scabies is often associated with complications caused by opportunistic or pathogenic bacteria, leading to rheumatic heart disease and/or acute streptococcal glomerulonephritis, either of which can be life threatening if left untreated. In animals, such as pigs, scabies is also important and causes increased maintenance costs, reduced growth rates, productivity losses, and major welfare issues [4]. Skin-to-skin contact and fomites are the key sources of transmission [1,5]. 

In the absence of a vaccine against scabies, drugs are presently the only therapeutic intervention option available. Systemic ivermectin and topical permethrin are the most commonly used compounds, but both of these drugs target the nervous system of the mite and are ineffective against the egg stage [6]. Repeated treatment with either of these drugs is required to suppress or eliminate infection, but their excessive and uncontrolled usage is contributing to the emergence of drug resistance issues [7]. Therefore, there is a need to search for a new intervention that targets the egg stage of *S. scabiei*. 

One possible approach to discovering novel intervention targets is to identify essential genes using functional genomic approaches, such as RNA interference (RNAi) [8]. Previous work [9,10] had identified 29 key genes in the RNAi pathway of *S. scabiei*, including those encoding homologues of *Exportin*, *Drosha*, *Dicer*, *Pasha*, *Loquacious*, *Argonaute*, *RdRP*, and *VIG*. Although it is not known whether this complement of genes/gene products is complete, RNAi has been shown to work reproducibly in female adults of *S. scabiei* [10] but has not been assessed on other developmental stages. As none of the currently approved drugs kill the egg stage of the mite [6], we have been eager to establish an RNAi method for this stage, in order to evaluate gene function and, importantly, to identify some key essential genes/gene products as potential intervention targets. The aims of the present study were to establish a practical, non-invasive method to deliver dsRNA into *S. scabiei* eggs and test the functionality of select single copy genes in *S. scabiei* eggs.

## 2. Results

Based on previous studies of mites [11,12] and in the absence of a model organism for *S. scabiei*, we were acutely aware of some challenges associated with the setting up of an RNAi method for mite eggs. First, we needed to ensure a ready supply of *S. scabiei* eggs (200 × 100 µm), which was achieved by yielding high numbers of them via our established pig–*S. scabiei* model [13]. Second, we needed to select genes amenable to RNAi. Third, we needed to ensure that we could efficiently deliver dsRNA through the eggshell to the developing embryo, achievable through validation/optimisation experiments. Fourth, we needed to assess the effect of egg pre-treatment and temperature on egg hatching. Fifth, we needed to demonstrate the reproducibility of experimental results. In the following, we addressed these key areas.

### 2.1. Selection of Genes

We selected three single copy genes of *S. scabiei*, designated *Ss-Cof*, *Ss-Ddp*, and *Ss-Nan*. Their homologs in *Drosophila melanogaster* had been shown to be essential [14,15,16,17,18,19], and transcription levels were high (2025 transcripts per million transcripts; *Ss-Cof*), moderate (602 TPM; *Ss-Ddp*), or low (373 TPM; *Ss-Nan*) in *S. scabiei* eggs, being 2.3-, 31-, and 186-times higher than transcription levels in female mites, respectively.

### 2.2. Efficiency of Delivery of dsRNA

**Figure 1:**
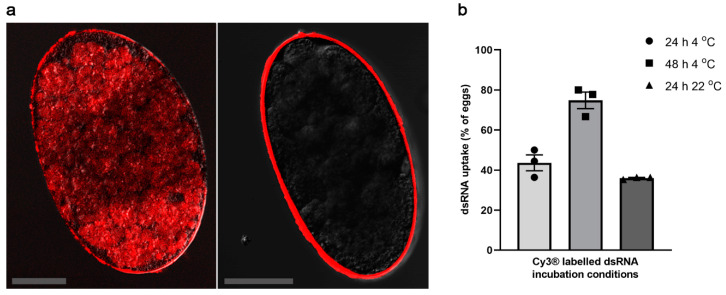
**dsRNA uptake by pre-treated *S. scabiei* eggs.** NaOCl pre-treatment facilitated the dsRNA delivery into *S. scabiei* eggs (**a**). Representative untreated egg that was incubated with Cy3^®^ labelled dsRNA shows no fluorescence signal inside the eggs (**a**; right) while a representative NaOCl pre-treated egg shows substantial fluorescence signal within the egg (**a**; left). Pre-treated eggs showed different efficiencies in Cy3^®^ labelled *Ec-LacZ* dsRNA uptake under different incubation conditions (**b**). Highest uptake was observed in 48 h 4 °C (**b**; ■) followed by 24 h 4 °C (**b**; ●) and 24 h at 22 °C (**b**; ▲). Scale bar = 50 µm. *n* = 3 × 20 eggs per group. Error bars represent mean ± the standard error of the mean (SEM).

Based on previous findings for tick eggs [20] and a series of pilot experiments, we elected to use NaOCl pre-treatment of *S. scabiei* eggs, directly followed by washing with physiological saline and immersion in dsRNA. We showed that NaOCl pre-treatment allowed the effective delivery of dsRNA into the eggs (Figure 1a; left), while none of the untreated eggs took up the fluorescently labelled *Ec-LacZ* dsRNA for any of the tested incubation conditions (Figure 1a; right). Depending on temperature and duration of incubation, NaOCl pre-treated eggs displayed different efficiencies in dsRNA uptake. The treatment of eggs with fluorescently labelled *Ec-LacZ* dsRNA for 48 h at 4 °C resulted in the highest number of eggs with positive fluorescence 74.8 ± 4.1% (mean ± SEM), whereas incubation for 24 h at 4 °C or room temperature (22 °C) showed 43.6 ± 4.0% or 36 ± 0.4% uptake, respectively (Figure 1b). Other conditions tested (NaOCl pre-treatment, followed by 1–4 h incubation at 37 °C, NaOCl treatment, followed by heat shock, electroporation only, pre-treatment with ethanol or pre-treatment with polybrene) did not result in adequate dsRNA-uptake (data not shown). Although NaOCl pre-treatment, followed by electroporation was suitable for delivering dsRNA, the egg recovery rate from the cuvette was very low and the procedure significantly reduced the hatching of larvae from eggs (data not shown). Hence, immersion for a longer incubation time at 4 °C was selected, in order to allow the measurement of transcriptional changes. 

### 2.3. Gene Knockdown by RNAi

**Figure 2:**
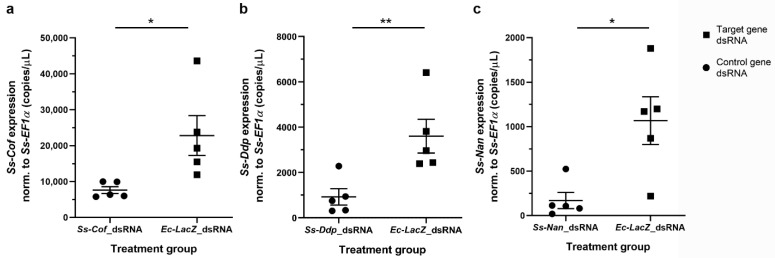
**Geneknockdown in *S. scabiei* eggs by RNAi.***Sarcoptes scabiei* eggs immersed in *Ss-Cof* (**a**), *Ss-Ddp* (**b**), or *Ss-Nan* (**c**) dsRNA showed a significant reduction (*p* < 0.05) in transcription compared with the *Ec-LacZ* dsRNA control under the same conditions. Transcription was normalised to the average level of *Ss-EF1α* transcription for all samples in each experiment. *n* = 5 × 25 eggs per group; error bars represent mean ± standard error of the mean (SEM); * *p* < 0.05; ** *p* < 0.01.

Having optimised dsRNA delivery, we proceeded to evaluate whether we could knockdown the genes *Ss-Cof*, *Ss-Ddp*, and *Ss-Nan* within eggs. The immersion of NaOCl pre-treated eggs in 2.5 µg/µL dsRNA of *Ss-Cof*, *Ss-Ddp* or *Ss-Nan* for 42 h at 4 °C, followed by 3 h at 37 °C resulted in a statistically significantly reduction of mRNA transcription (*p* = 0.0256, *p* = 0.0095, and *p* = 0.0128, respectively), compared with *Ec-lacZ* dsRNA-immersed eggs (Figure 2, Table S1). Mean reductions in transcription for the genes *Ss-Cof*, *Ss-Ddp*, and *Ss-Nan* relative to the *Ec-lacZ* control were 66.6% (mean of difference (MD) = 15,200 copies per µL; standard deviation (SD) = 11,500 copies per µL), 74.3% (MD = 2670 copies per µL; SD = 1700 copies per µL), and 84.1% (MD = 898 copies per µL; SD = 579 copies per µL), respectively.

### 2.4. Effect of NaOCl Pre-Treatment and Temperature on ‘Egg Hatching’

**Figure 3:**
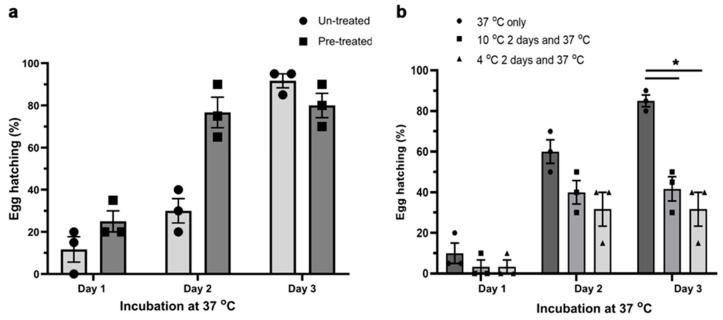
**Effect of NaOCl pre-treatment and incubation temperature on the hatching of larvae from *S. scabiei* eggs in vitro.** NaOCl pre-treatment did not alter the physiological hatching time of *S. scabiei* eggs (**a**). There was no statistical difference in hatching between eggs that were pre-treated with NaOCl and those that were not (**a**, day 3). However, incubation temperature did have a significant effect on the hatching (**b**). The optimum temperature for untreated eggs was 37 °C, whereas 2 day periods of 10 °C (*p* = 0.0169) or 4 °C (*p* = 0.0297) incubation prior to 37 °C incubation, to allow optimal dsRNA uptake, led to a significant reduction in the hatchability (**b**, day 3). *n* = 3 × 20 eggs per group; error bars represent mean ± standard error of the mean (SEM); * *p* < 0.05.

We critically assessed whether NaOCl pre-treatment and temperature affected the emergence of *S. scabiei* larvae from the dsRNA-treated eggs. On day 3 (37 °C incubation), which is known as the optimal hatching temperature for eggs of *S. scabiei* [5], no significant difference in hatching was observed between eggs pre-treated with 2% NaOCl and those that were not (Figure 3a). However, most of the pre-treated eggs had hatched by day 2. In addition, although low incubation temperatures improved the delivery of dsRNA into eggs (Figure 1b), low temperatures (10 °C (*p* = 0.0169) and 4 °C (*p* = 0.0297)) affected the egg hatching significantly (Figure 3b) compared with hatching at 37 °C, showing that 37 °C was the optimum temperature for hatching.

### 2.5. Assessing Phenotypic Alterations following RNAi

**Figure 4:**
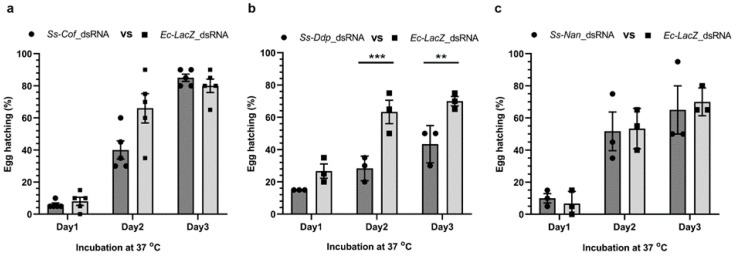
**Effects on the hatchability of *S. scabiei* eggs following treatment with dsRNA.** Egg hatchability was monitored at 37 °C for 3 days following incubation with *Ss-Cof* (**a**), *Ss-Ddp* (**b**), or *Ss-Nan* (**c**) dsRNA at room temperature (22 °C) for 24 h. There was significant reduction in larval hatching from eggs following silencing with *Ss-Ddp* (**b**) compared with the *Ec-LacZ* control (*p* = 0.0009 at day 2 and *p* = 0.0070 at day 3). There was no significant change in hatchability for the other two treatment groups (**a**,**c**). *n* = 3 × 20 or 5 × 20 eggs per group; error bars represent mean ± standard error of the mean (SEM); ** *p* < 0.01; *** *p* < 0.001.

Although the low incubation temperature (4 °C) enhanced dsRNA uptake into *S. scabiei* eggs (Figure 1b), it inhibited the development of the larvae within the egg and/or larval hatching from the egg (Figure 3b). Therefore, experiments to observe RNAi-induced phenotypic changes were conducted using increased incubation temperatures (22 °C for 24 h followed by 37 °C for 3 days). Under these conditions, pre-treated eggs were incubated with 2.5 µg/µL dsRNA in physiological saline for 24 h at room temperature (22 °C), followed by 3 days at 37 °C in a humidity chamber to assess the phenotypic effects of gene silencing on each of the three target genes. There was no significant effect on the hatchability incubated in *Ss-Cof* (*p* = 0.0743) or *Ss-Nan* (*p* = 0.9889) dsRNA compared with the *Ec-LacZ* control (Figure 4a,c). However, at the end of the incubation (day 3 at 37 °C), there was a significant reduction in hatchability (mean: 38.1%; *p* = 0.0070) of the eggs incubated in *Ss-Ddp* dsRNA compared with the *Ec-LacZ* dsRNA control eggs (Figure 4b). 

## 3. Discussion

This study provides proof of principle and establishes an RNAi-based tool to study the function(s) of genes and/or gene products in the egg stage of *S. scabiei*. Prior to this study, RNAi experiments in mite eggs were conducted in the pest mites *Tetranychus urticae* and *Panonychus citri* via the vertical delivery of dsRNA [12,21]—i.e., by treating gravid females which transferred the RNA to unlaid eggs. With no available in vitro culture system, this method is not feasible for *S. scabiei*, as oviposition is compromised when this mite is maintained outside of its host environment. In ticks (which are related to mites), dewaxing or electroporation was used to permeabilise the egg shell and deliver the dsRNA into eggs [20,22], but hatchability was significantly compromised using these methods. *Sarcoptes scabiei* eggs are naturally impermeable to the dsRNA (Figure 1a, right), in accordance with observations for the eggs of a related mite, *Psoroptes ovis* [11]. Therefore, we pre-treated *S. scabiei* eggs with NaOCl; pre-treatment with 2% NaOCl for 25 s resulted in a dsRNA-uptake by 75% of the eggs within 48 h at 4 °C. This pre-treatment did not affect the larval hatching from eggs (Figure 3a). Thus, this non-invasive, yet effective dsRNA delivery method was suited to functional genomic investigations of eggs and the discovery of essential genes as potential drug or vaccine targets. 

We elected to specifically target single copy genes, each encoding a single protein, in order to ensure specific binding of dsRNA to the target gene. The level of transcription of each gene was also considered, and three target genes of high (*Ss-Cof*), medium (*Ss-Ddp*), and low (*Ss-Nan*) transcription level were selected. It was encouraging to observe that RNAi worked irrespective of transcription level. Finally, we selected target genes according to the essentiality of homologues in *D. melanogaster* and phenotypes upon knockdown. In *Drosophila*, the *Cofilin* gene controls the length and dynamic rearrangement of the actin filaments and oogenesis [14,15]; the *Deadpan* gene is predicted to have a positive regulatory effect on neurogenesis, neuroblast proliferation, sex determination, establishment of X:A ratio, and locomotor behaviour [16,17]; and the *Nanos* gene is involved in cell development, oogenesis, segmentation, and spermatogenesis [18,19]. Based on this information, we expected to observe a phenotypic effect of gene knockdown for each of these genes, but confocal microscopic observations showed no differences between treated and untreated eggs (data not shown). Changes in ‘egg hatchability’ were observed for only one of the three genes, although silencing was proven for each gene by a reduction in transcription, detected by specific qPCR. 

Environmental temperature was inversely related to dsRNA-uptake by *S. scabiei* egg and directly proportional to hatching. This was in accord with a previous study, where *S. scabiei* eggs pre-incubated at lower temperatures exhibited partial or complete inhibition of hatching in vitro [5]. This hindered the observation of a phenotype at the highest dsRNA-uptake, followed by highest efficiency in gene silencing. 

Silencing genes using siRNAs or dsRNAs have been successful in mites [10,11,12,23,24,25], but dsRNAs have been predominantly used in ticks—which have ability to naturally generate siRNA with high specificity and efficiency [26,27,28]. To our knowledge, it has not been assessed experimentally whether mites naturally produce siRNA efficiently. Even though dsRNA has been more frequently used in mites, varying efficacies of gene silencing have been reported among species for the same target genes; dsRNA was most efficient in silencing the *GST-mu1* in *Dermatophagoides pteronyssinus* [23], and *vATPase* subunit A in the poultry red mite *Dermanyssus gallinae* [29], while siRNA was the most efficient in silencing the *distal-less* gene of the spider mite *T. urticae* [12]. Nevertheless, we chose to use dsRNA in *S. scabiei* eggs, aiming to achieve better silencing with endogenously produced siRNA.

Establishing an RNA interference tool to silence genes in *S. scabiei* eggs provides a unique opportunity to study the function of genes on a broader scale, and to identify essential genes as novel intervention targets. This is of particular importance, given that mostly commonly used drugs do not kill eggs in *S. scabiei*-infested skin. This tool could also be applied to study early embryo development, and might find application to other parasitic mites in functional genomic investigations

## 4. Materials and Methods

### 4.1. Procurement of S. scabiei Eggs

*S. scabiei* var. *suis* eggs were sourced from experimentally infected pigs [13]. Skin crusts from infected pigs containing all of the developmental stages (i.e., eggs, larvae, nymphs, and adults) were collected [30], and eggs at the blastula stage or younger isolated within 5 h of sampling. 

### 4.2. Selection of Single-Copy Genes and Synthesis of dsRNA

Here, we selected three single copy genes from the *S. scabiei* genome. To do this, we first identified all paralogous gene groups using the program OrthoMCL v2.0.4 [31] employing a cut-off E-value of 1 × 10^−6^, and then identified individual single-copy genes that were not in any such a group. From a total of 5139 single-copy genes, three such genes with ‘low’, ‘medium’, or ‘high’ transcription in eggs of *S. scabiei* (based on transcripts per million, TPM) and with homologues of known function in *D. melanogaster* were ultimately selected for RNAi.

Then, dsRNA was synthesised from the each of these genes—i.e., *Ss-Cof* (GenBank KAF7491759.1), *Ss-Ddp* (GenBank KAF7493355.1), and *Ss-Nan* (GenBank KPM04769.1)—and from the *LacZ* gene of *Escherichia coli* strain K-12 sub-strain MG1655 (GenBank NC_000913.3) as an ‘irrelevant’ (negative) dsRNA control. Primer pairs to amplify partial sequences of the three target genes were designed using the Primer 3 program [32], and specifically amplified gene fragments were cloned into the dsRNA production vector pL4440 at respective restriction sites. An *E. coli LacZ* clone [10] was used to generate negative control dsRNA. The list of primers, target sequence lengths, and restriction site enzymes used for directional cloning are given in Table 1. Plasmids were cleaved with *Sac*I or *Xba*I (NEB, Ipswich, MA, USA) and then treated with Klenow enzyme (NEB, Ipswich, MA, USA) to remove overhangs. The T7 RiboMAX™ Express RNAi system (Promega, Madison, WI, USA) was used to produce dsRNA following the manufacturer’s instructions; dsRNA pellets were dissolved in 0.9% nuclease-free NaCl. ND1000^®^ a Nano-drop spectrophotometer (Thermo Fischer Scientific, Waltham, MA, USA) was used for dsRNA quantification, and agarose gel electrophoresis was used to assess the dsRNA quality. 

### 4.3. NaOCl Pre-Treatment of Eggs 

For each treatment group, ~80 *S. scabiei* eggs (containing blastomere or earlier stage) were collected on a stainless steel wire mesh (77 µm aperture; Advanced Engineering Group, Wacol, QLD, Australia) and immersed in 20 µL nuclease-free 0.9% NaCl (Sigma Aldrich, Saint Louis, MO, USA) on a detached 1.5 mL microcentrifuge tube cap. To render the egg shell permeable to dsRNA in normal saline, the eggs were pre-treated by dipping the mesh containing the eggs into 20 µL of 0.2% nuclease free NaOCl (Sigma Aldrich, USA) for 25 s, followed by three 25 s wash steps each time using 20 µL of sterile, nuclease-free 0.9% NaCl (Sigma Aldrich, Saint Louis, MO, USA). 

### 4.4. Transfer of dsRNA to Eggs

*Ec-LacZ* dsRNA was labelled with the dye Cy3^®^ [10] using the Silencer™ siRNA labelling kit (Thermo Fischer Scientific, Waltham, MA, USA). Twenty-five each of pre-treated or untreated eggs were incubated in 2.5 µg/µL of Cy3^®^ labelled *Ec-LacZ* dsRNA in 0.9% nuclease free NaCl at 4 °C for 24 or 48 h or at room temperature (22 °C) for 24 h. Incubated eggs were washed by dipping them 3 times into PBS-0.05% Tween (PBST) and once into PBS to remove excess dsRNA before examination under a Zeiss 780-NLO confocal microscope with a 20× Plan-Apochromat (NA 0.8) lens. Z stack images (4–8 slices) were captured. Cy3^®^ was excited using a 561 nm laser attenuated 97% and emitted light (565–631 nm) was captured using a GaAsP array detector. Images are presented as a single representative slice through each egg processed using Zen software (Carl Zeiss AG, Oberkochen, Germany). Eggs with developed larvae were excluded, to eliminate false positive signals due to autofluorescence of the *S. scabiei* cuticle. The dsRNA uptake efficiency was determined as the percentage of fluorescent positive eggs of the total number of eggs incubated. 

### 4.5. Evaluation of Transcriptional Knockdown

Five replicates of 25 pre-treated eggs were completely immersed (as described above) in 20 µL of 2.5 µg/µL *Ss-Cof* or *Ss-Ddp* or *Ss-Nan* or negative control *Ec-LacZ* dsRNA preparations and closed caps were incubated at 4 °C for 42 h. The eggs were then incubated at 37 °C for 3 h (essentially physiological conditions) before RNA extraction; dsRNA in solution was removed and eggs were washed three times with 0.9% nuclease free NaCl. Total RNA was extracted in 10 µL nuclease-free water [10] using the Quick-RNA™ Tissue/Insect Microprep kit (Zymo Research, Irvine, CA, USA). The total RNA extracted was used to make cDNA using the Superscript^®^ II reverse transcriptase (Thermo Fischer Scientific, Waltham, MA, USA) and Oligo(dT) 20 primer (Thermo Fischer Scientific, Waltham, MA, USA). Final concentrations of the reaction mix were 1.25 µM Oligo(dT) 20 primer, 200 U Superscript^®^ II reverse transcriptase, first strand buffer to 1X, RNase, and 10 mM dithiothreitol (DTT) in a 20 µL reaction. Briefly, RNA and Oligo(dT)20 primer were denatured at 65 °C for 5 min and placed on ice. First-strand buffer, RNase, and DTT were added and incubated at 42 °C for 2 min incubation. Superscript^®^ II was added and incubated at 42 °C for 50 min to synthesise cDNA, and enzymes were inactivated at 72 °C for 15 min before storage at −20 °C. 

To assess gene knockdown, the transcription of each gene was assessed by quantitative PCR using gene-specific primers designed using Primer 3 [32], excluding the dsRNA-targeted sequence (Table 1). The constitutively transcribed housekeeping gene *elongation factor 1 alpha* (*Ss-EF1α*) of *S. scabiei* [33] was used to normalise transcription. A qPCR (10 µL) contained LightCycler^®^ 480 SYBR Green I Mater (Roche, Millers Point NSW, Australia) to 1×, 0.5 µM each primer and 1–3 µL of cDNA (i.e., 1 µL for highly expressed *Ss-Cof*, 2 µL for *Ss-Ddp*, and 3 µL for *Ss-Nan*). Transcription was quantified (in triplicate) using the CFX384 Touch™ Real-Time PCR detection system (Bio-Rad, Hercules, CA, USA) and the following cycling conditions: 95 °C for 10 min, followed by 45 cycles of 95 °C for 10 s, 57 °C (*Ss-Cof*, *Ss-Ddp* and *Ss-Nan* primers) or 50 °C (*Ss-EF1α* primers) for 10 s, and 72 °C for 10 s. Conditions for melt curve analysis were 95 °C for 5 s, 65 °C for 5 s, and 95 °C for 5 s. To prepare qPCR standards, each qPCR target gene fragment was cloned into pMiniT vector (NEB, Ipswich, MA, USA) following the manufacturer’s instructions for PCR cloning kit (NEB, Ipswich, MA, USA). Copy number for each clone was calculated using DNA copy number calculator online tool (Thermo Fischer Scientific, Waltham, MA, USA) and dilutions were prepared for 10^2^–10^9^ copies/µL. Student’s *t*-test (GraphPad Software Inc., San Diego, CA, USA) was used to calculate the statistical differences of the normalised average gene transcription between treatment and control groups; *p <* 0.05 was considered as significant.

### 4.6. Determining the Effects of NaOCl Pre-Treatment on Egg Hatching

Pre-treated or untreated eggs were randomly assigned to three replicates of 20 eggs each and submerged in 20 µL of 0.9% NaCl on detached 1.5 mL microcentrifuge tube caps, and the caps were closed with the detached tube. The eggs were incubated at 37 °C for three days in a humidified container, and the total number of hatched larvae was recorded each day (i.e., every 24 h). The statistical difference in hatch rate between treated and untreated eggs on individual days was calculated using the two-way ANOVA method with multiple comparison in GraphPad Prism 8 (GraphPad Software Inc., San Diego, CA, USA); *p* < 0.05 was considered as statistically significant. 

### 4.7. Determining the Effects of Low Temperature on Hatching of Larvae from Eggs

To determine the effect of incubation temperature on ‘egg hatching’, untreated eggs were incubated for 3 days at 37 °C or 2 days at 4 °C, followed by 3 days at 37 °C or 2 days at 10 °C followed by 3 days at 37 °C in 20 µL of nuclease free 0.9% NaCl placed on closed microcentrifuge caps (as described above). Eggs were monitored, and hatching was recorded each day (i.e., every 24 h; 37 °C incubation). The statistical difference in hatch rate at different incubation temperatures on individual days was calculated and total numbers of larvae that hatched on individual days were used separately for analysis. Results were analysed by two-way ANOVA with multiple comparison using GraphPad Prism 8 (Graph-Pad Software Inc., San Diego, CA, USA); and *p* < 0.05 considered statistically significant.

### 4.8. Assessing Morphological Alterations in dsRNA-Treated Eggs

Three replicates of 20 NaOCl pre-treated eggs were separately immersed in 20 µL of 2.5 µg/µL *Ss-Cof* or *Ss-Ddp* or *Ss-Nan* target gene dsRNA or negative control *Ec-LacZ* dsRNA on microcentrifuge tube caps. The caps were closed and incubated in a humidified chamber for 24 h at room temperature (22 °C) and 3 days at 37 °C. Hatching was recorded every 24 h, and the difference in hatch rate between treatment and control groups assessed at each 24 h interval analysed by two-way ANOVA with multiple comparisons (GraphPad Software Inc., San Diego, CA, USA), defining *p* < 0.05 as statistically significant. The same approach was used to appraise unhatched embryos for possible morphological alterations. Ten unhatched eggs or larvae of every treatment or control group in every 24 h interval were examined by confocal microscopy as described, except that photomultiplier for transmitted light was used instead of a fluorescence detector. 

## Figures and Tables

**Table 1:** Primer sequences with restriction site enzymes and respective target gene length.

Gene		Primer Sequences	Restriction Enzyme Site	Encoding Sequence Length (bp)
**Primers for dsRNA**	*Ss-Cof*	F—5′-TAT GAG CTC AAC AAC TGG ACC TCG TGA CT-3′	*Sac*I	185
		R—5′-TAT TCT AGA GCA CTT TCC GGG CAC-3′	*Xba*I	
	*Ss-Ddp*	F—5′-TAT GAG CTC ATA TCG TGC CGG CTT TTC TG-3′	*Sac*I	226
		R—5′-TAT CCC GGG ATG AAG AGT GTT GGC GTT CG-3′	*Sma*I	
	*Ss-Nan*	F—5′-TAT GAG CTC TGC CAT CGA AAC CTA TAC CCA-3′	*Sac*I	209
		R—5′-TAT CCC GGG GGT CGC ATC ACA GAT AG-3′	*Sma*I	
	*Ec-LacZ*	F—5′-TAT GAG CTC CGT TAC CCA ACT TAA TCG CC-3′	*Sac*I	319
		R—5′-TAT CCC GGG TGT GAG CGA GTA ACA ACC C-3′	*Sma*I	
**Primers for qPCR**	*Ss-Cof*	F—5′-AAG CGC CAT GAG TGT TGA TC-3′		161
		R—5′-CTG AAG CTT CCT CAT AAT CGC A-3′		
	*Ss-Ddp*	F—5′-ATC ATT GCG TCA ACA GCC TC-3′		167
		R—5′-TTG CGG ATT GAT TGA GCC AG-3′		
	*Ss-Nan*	F—5′-TCG ATG TAT TGT GGC TTC TGT-3′		187
		R—5′-TCC CGT TCA ATG TAG TCC GT-3′		
	*Ss-EF1* *α*	F—5′-TTG GCT TAT ACC TTG GGT GTG-3′		181
		R—5′-CAC CGT TCC ATC CAG AGA TT-3′	na	

na—not applicable; F—forward primer; R—reverse primer.

## Data Availability

All data from this study are available within this article.

## Supplementary Materials

The following supporting information can be downloaded at: https://www.mdpi.com/article/10.3390/ijms23020873/s1.
